# Qualitative Interview Analysis: Unpacking Packed Interviews

**DOI:** 10.1093/geroni/igaa057.136

**Published:** 2020-12-16

**Authors:** Michelle Silver

**Affiliations:** University of Toronto, Toronto, Ontario, Canada

## Abstract

Retirement is an ever-evolving, dynamic, and complex social construct we associate with the end of one’s career. Exploring what retirement means to different people can contribute to a better understanding of the implications of this important transition at the individual and societal level. However, sifting through participants stories is not always a straightforward endeavor, particularly in the case when participants have something to hide. This paper examines the value of qualitative research methods in unpacking complex personal narratives. As the landscape surrounding mature workers’ experiences continues to change, this paper extends policy debates about retirement, as well as scholarly conversations about the richness and complexity of qualitative research.

